# Effects of Grinding Parameters on the Processing Temperature, Crack Propagation and Residual Stress in Silicon Nitride Ceramics

**DOI:** 10.3390/mi14030666

**Published:** 2023-03-16

**Authors:** Haipeng Yan, Fei Deng, Zhiying Qin, Jinda Zhu, Hongjie Chang, Huli Niu

**Affiliations:** School of Mechanical Engineering, Hebei University of Science and Technology, Shijiazhuang 050018, China; yanhp@hebust.edu.cn (H.Y.);

**Keywords:** silicon nitride ceramics, surface quality, crack propagation, residual stress, grinding

## Abstract

The surface/subsurface damage of engineering ceramics after machining has a great influence on the service performance of parts. In order to obtain a high grinding surface quality of engineering ceramics, and take silicon nitride ceramic as a research object, a series of grinding experiments were carried out. The effects of grinding parameters on longitudinal crack propagation depth and the surface residual stress of silicon nitride ceramics were analyzed by grinding experiments, and the residual stress at the location of crack propagation was obtained. The variation in the grinding temperature under different grinding parameters was explored. The influences of the grinding temperature on crack propagation depth and surface residual stress were clarified, the distribution of residual stress along the depth direction was discussed, and the relationship between the residual stress and crack propagation was revealed. The results show that the residual compressive stress on the surface of silicon nitride ceramics decreases with the increase in the depth of crack propagation and the degree of surface brittle spalling. The residual stress at the location of the crack propagation was residual tensile stress. The crack propagation depth increased with the increase in the residual tensile stress. The research provides a reference for the realization of high-quality surfaces in the grinding of silicon nitride ceramics.

## 1. Introduction

Engineering ceramics represented by silicon nitride (Si_3_N_4_) ceramics are widely used in precision machinery, electrics and electronics, military equipment, and aerospace because of their excellent characteristics such as high strength, high hardness, wear resistance, corrosion resistance, low density, and insulation [1,2,3,4]. Engineering ceramics, as a difficult-to-machine material, have poor machinability [5]. Because of their hard and brittle characteristics, grinding has become the main processing method for ceramic parts [6,7]. In the process of grinding, high temperature, grinding wheel wear, surface burn, crack generation and crack propagation are very easy to occur, which not only reduces the service life of the grinding wheel but also seriously affects the processing quality, accuracy, and efficiency [8].

During the grinding process, the surface material of the workpiece is subject to shear slip, severe friction, high temperature, and high pressure, which causes severe plastic deformation, and thus generates residual stress on the surface of the workpiece [9]. The residual stress will directly affect the fracture stress, bending strength, fatigue strength, and corrosion resistance of engineering ceramic parts [10]. Engineering ceramics are typically hard and brittle engineering materials. The fracture stress and toughness of ceramic parts are more sensitive to the surface stress state than that of metals [11]. Whether the residual compressive stress or tensile stress has an important impact on the fracture toughness of the part, especially on the generation and propagation of the crack, remains to be seen [12]. The cracks in the surface/subsurface of parts greatly affect their performance and service life, so it is particularly important to explore the relationship between residual stress and crack growth of engineering ceramics.

In recent years, researchers have conducted a lot of studies on material removal mechanisms, grinding temperatures, residual stress, and the crack growth of engineering ceramics from the aspects of machining methods, wheel grain size, grinding parameters, etc. Dai et al. [13] researched the damage formation mechanism of sintered silicon carbide through a single-diamond grinding experiment. The results showed that it had four stages, including purely ductile, primarily ductile, primarily brittle, and purely brittle, in the single-diamond grinding process of sintered silicon carbide. Wang et al. [14] established the molecular dynamics simulation model of nano-cutting single-crystal tungsten and analyzed the effects of tool geometry on material removal behavior. The research indicated that a larger positive rake angle, or larger clearance angle or smaller edge radius of the tool could improve surface quality. Further, Wang et al. [15] discussed the effects of the cutting force on the subsurface damage of tungsten and found that the variation in the cutting force had a great influence on the number of surface defect atoms, dislocation density, and the thickness of the subsurface damage layer. Du et al. [16] studied the surface quality and residual stress of alumina ceramic and zirconia ceramic after grinding under preloading stress. The surface damage of ceramics caused by abrasive grinding was reduced by using the method of preloading stress grinding. The relationship between surface residual stress, grinding depth and preloading stress was obtained. Sun et al. [17] explored the crack-extending mechanism of silicon nitride ceramics in grinding and obtained the relationship between the crack growth and grinding parameters. Denkena et al. [18] presented an innovative quick-stop device for grinding mixed oxide ceramics. The study obtained the influence of grinding parameters on the number of effective abrasive particles involved in grinding, grinding force, maximum undeformed chip thickness, and grinding contact surface temperature. Ma et al. [19,20] carried out the laser-assisted grinding of zirconia ceramic materials, studied the influence of the grinding temperature on the depth of brittle plastic transition of materials, and analyzed the grinding surface integrity and compared it with the traditional method. Those studies found that laser-assisted grinding could significantly improve the surface quality of materials. Fergani et al. [21] put forward a physical model in the experiment of grinding temperature and residual stress. The model could predict the initial temperature as a function of residual stress on a quantitative basis. It could analyze and calculate the thermal stress using Timoshenko’s thermal stress theory, and the residual stress distribution could be predicted under given process conditions and material properties through grinding experiments. Wan. et al. [22] investigated the grinding parameters on the subsurface damage, residual stress, and grinding temperature in the grinding of zirconia ceramic by a thermal-mechanical coupling model based on the finite element method. It was concluded that the depth of the subsurface damage could be reduced by controlling reasonable grinding parameters. Wu et al. [23] found that a proper grinding temperature can inhibit crack growth in grinding silicon nitride ceramics experiments and proposed that grinding temperature and crack growth could be properly controlled by constant speed ratio grinding.

High-quality grinding target factors include machining accuracy and surface quality after machining. As far as the surface quality after machining is concerned, it includes multiple evaluation indicators such as surface roughness, surface waviness, surface hot cracks, residual stress, and the surface metamorphic layer [24]. The above research shows that the exploration of residual stress and crack growth in the grinding of engineering ceramics was of great significance to the surface integrity of its workpiece. However, previous studies only focused on the grinding residual stress or crack growth of engineering ceramics, and the relationship between the two was not analyzed. In this paper, the residual stress and crack growth in the grinding of silicon nitride ceramics were studied. The influences of grinding parameters on residual stress, crack propagation, and grinding temperature were discussed based on a series of experimental results. The effect of the grinding temperature on residual stress and crack propagation depth was researched. The relationship between residual stress and crack propagation was revealed.

## 2. Materials and Methods

### 2.1. Experiment for Crack Propagation Analysis

**Figure 1:**
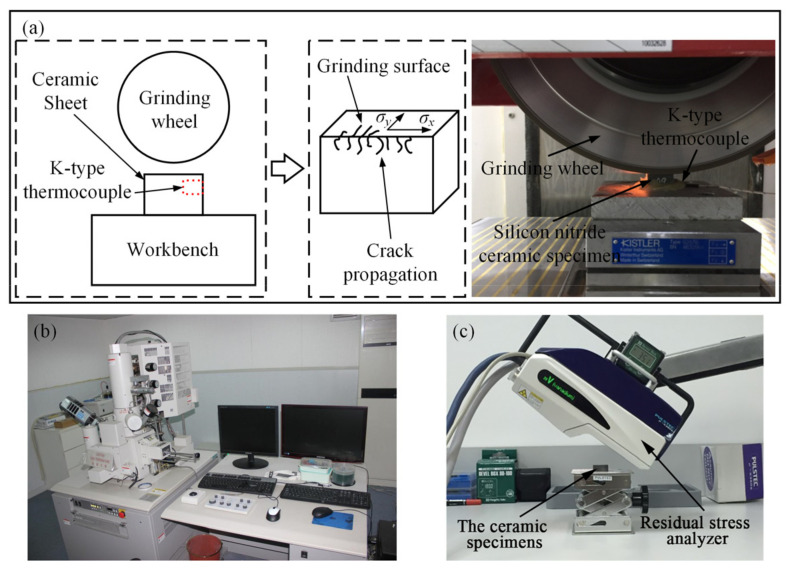
Grinding experiment and measuring equipment: (**a**) Experimental principle and processing; (**b**) SEM; (**c**) Residual stress analyzer.

The specimens were hot isostatically pressed silicon nitride (HIPSN) ceramic with the size of 20 mm × 20 mm × 3 mm manufactured by Beijing Sinoma Synthetic Crystal Co. Ltd., Beijing, China, and the material properties [23] were listed in Table 1. As shown in Figure 1, a ceramic sheet was clamped with special fixtures and fixed on the table to grind the surface of 20 mm × 3 mm. The surface and sub-surface crack propagation could be observed more conveniently by selecting the flaky ceramic for experiments. The machine tool for grinding was BLOHM Orbit 36 precision planar forming grinder manufactured by Korber Schleifring, Hamburg, Germany. The grinding wheel was an artificial diamond wheel (D91, diameter of 200 mm, width of 20 mm). The grinding temperature was measured by a K-type thermocouple. One end of the thermocouple was fixed at a workpiece side surface 2 mm below the grinding surface, and the other end was connected to a multi-channel temperature measuring instrument for collecting temperature data. The surface cracks of specimens after machining were observed by Hitachi S-4800 cold field emission scanning electron microscope (SEM) (Hitachi, Tokyo, Japan). The experiments were carried out under the condition of dry grinding. Dry grinding can avoid the cooling fluid from entering the grinding area and affecting the measured value of temperature for the contact measurement method. A series of crack propagation in the surface/subsurface of the specimens under different grinding conditions could be obtained by adjusting grinding parameters (wheel speed *v_s_*, grinding depth *a_p_*, and feed rate *v_w_*). As silicon nitride ceramics are processed by hot isostatic pressing sintering, they have compactness, small grain size, and high grain refinement. Therefore, there is no micro-crack on the surface, which is relatively smooth. However, due to the deviation of the sintering process (holding time and pressure application), there are very few pores on the surface of the material, which do not affect the performance analysis of the material after processing.

### 2.2. Experimental Equipment for Residual Stress Analysis

A portable X-ray residual stress analyzer μ-x360s (Pulstec, Hamamatsu, Japan) was used to measure the residual stress on the surface of ceramic materials after grinding. As shown in Figure 1c, this analyzer could detect the residual stress of the measured specimen quickly and accurately. Based on crystal diffraction and Hooke’s law, the residual stress on the surface and inside of the measured workpiece could be determined according to the change in the interplanar spacing of the material.

The residual stress values under different conditions were obtained by changing the grinding parameters. The experiments are consistent with that Section 2.1. Due to the hardness and brittleness of ceramic materials, there are mainly two removal methods in grinding. They are plastic deformation and brittle fracture. Additionally, these two removal methods lead to a large difference in the residual stress states of ceramic materials [5]. In addition, the original stress σ on the surface of the specimen without grinding was related to the sintering process of the material. The original residual stress on the surface of the silicon nitride ceramic specimen was measured as *σ_x_*_0_ = −146 MPa and *σ_y_*_0_ = −79 MPa before the experiment. It could be found that the original residual stress of the silicon nitride ceramic was compressive stress. However, the values in the direction parallel to the grinding direction (*σ_x_*) and perpendicular to the grinding direction (*σ_y_*) were different. There are both influences of different initial stress values on abrasive cutting and crack growth during grinding, which may affect the final experimental results. Therefore, the measured values in the experiments are relative values obtained on the basis of the original residual stress. In order to ensure the accuracy of the experimental results, multi-point tests were conducted on the ground surface of workpieces to take the average value.

## 3. Results and Analysis

### 3.1. Influence of Grinding Parameters on Crack Propagation

**Figure 2:**
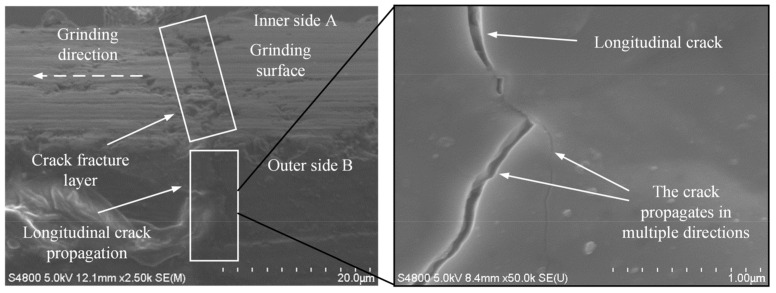
Details of crack propagation (*v_s_* = 38 m/s, *a_p_* = 20 μm, *v_w_* = 3500 mm/min).

Figure 2 displays the observation results of the surface/subsurface of silicon nitride ceramic after grinding. It can be seen from Figure 2 that hot cracks appeared on the surface/subsurface of silicon nitride ceramics. Since the thickness of ceramic sheets is far less than the grinding width of the grinding wheel, the surface/subsurface is easy to be crossed by the cracks from side A to side B in the direction perpendicular to the grinding direction. In addition, under the combined action of grinding heat and grinding force, a fractured layer appeared on the ground surface, which led to an inconsistent height on both sides of the crack. Due to the large grinding depth and high feed rate during the grinding process, the grinding temperature and grinding force were both large. Moreover, when the value of maximum undeformed chip thickness *h_max_* (Equation (1)) [25] was actually cut by abrasive grains in the grinding, it exceeded the critical cut depth *h_c_* (Equation (2)) [26] of the ductile-brittle transition of ceramic materials, and the material removal dominated in brittle mode, which made it easy to produce crack. According to Equations (1) and (2), the value of the critical cut depth for silicon nitride ceramic was calculated as 0.05 μm, and the value of the maximum undeformed chip thickness under present grinding parameters was a value of 0.41 μm to 1.07 μm. Thus, it caused the brittle fracture of ceramic materials and produced microcracks. It can be found in the observation that cracks on the subsurface of the workpiece after grinding mainly existed in the form of longitudinal cracks. The cracks gradually extended to the interior of the ceramic material in multiple directions, resulting in subsurface damage. However, it did not cause the macroscopic fracture of the ceramic specimen.

(1) $$h_{\max}={\left(\frac{3}{N_{s}\tan\alpha }\right)}^{1/2}{\left(\frac{v_{w}}{v_{s}}\right)}^{1/2}{\left(\frac{a_{p}}{d_{s}}\right)}^{1/4}$$

(2) $$h_{c}=\beta \left(\frac{E}{H}\right){\left(\frac{K_{IC}}{H}\right)}^{2}$$

where *N_s_* is the active number of abrasive grains involved in grinding, *α* is the semi-angle of the undeformed chip cross-section, *d_s_* represents the diameter of the grinding wheel; *v*_s_ represents the wheel speed; *a_p_* represents grinding depth; *v_w_* represents the feed rate, *β* is the coefficient of processing condition, *E* is the elastic modulus of the material, *H* is material hardness, and *K_IC_* is the fracture toughness of ceramic materials.

**Figure 3:**
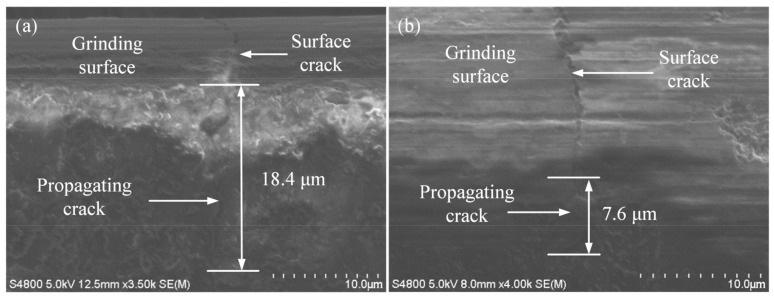
Influence of wheel speed on crack propagation (*a_p_* = 12 μm, *v_w_* = 3500 mm/min): (**a**) *v_s_* = 30 m/s; (**b**) *v_s_* = 40 m/s.

**Figure 4:**
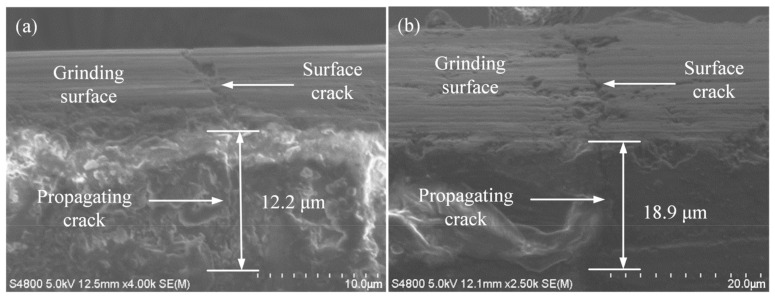
Influence of grinding depth on crack propagation (*v_s_* = 38 m/s, *v_w_* = 3500 mm/min): (**a**) *a_p_* = 10 μm; (**b**) *a_p_* = 20 μm.

**Figure 5:**
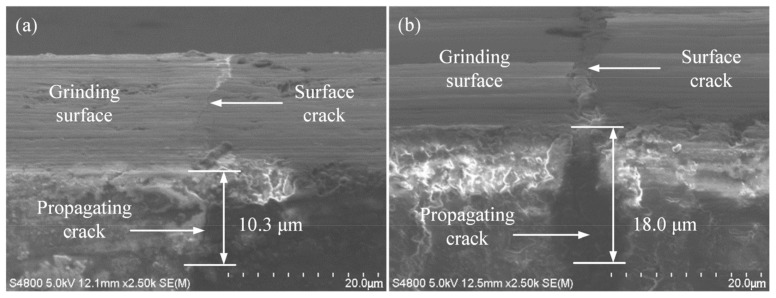
Influence of feed rate on crack propagation (*v_s_* = 38 m/s, *a_p_* = 12 μm): (**a**) *v_w_* = 2500 mm/min; (**b**) *v_w_* = 5500 mm/min.

As shown in Figure 3, Figure 4 and Figure 5, some microcracks were produced in the surface/subsurface during the grinding process with the brittle removal material.

Figure 3 shows the relationship between the wheel speed vs. and the crack propagation depth *h*. It can be seen from Figure 3 that when the grinding depth was 12 μm, the feed rate was 3500 mm/min, and the wheel speed was 30 m/s, the longitudinal crack propagation depth was 18.4 μm. When the wheel speed was 40 m/s and the crack propagation depth was 7.6 μm. It can be known that increasing the wheel speed decreases the crack propagation depth. The reason for this phenomenon may be that the increase in wheel speed increased the number of effective abrasive grains that participated in grinding per unit of time, which increased the heat flux density in the grinding area [27]. In addition, an increase in wheel speed forms an air barrier in the grinding area, which obstructs the external convection heat transfer and leads to the temperature rise in the grinding area. An appropriate high temperature of grinding increases the value of the fracture toughness of silicon nitride ceramics, which can increase the critical cutting depth of its ductile-brittle transition [28]. It also increases the proportion of the plastic removal of materials, thus inhibiting the generation and propagation of cracks.

Figure 4 shows the relationship between the grinding depth *a_p_* and the crack propagation depth *h*. It can be seen from Figure 4 that when the wheel speed was 38 m/s, the feed rate was 3500 mm/min, the grinding depth was 10 µm, and the longitudinal crack propagation depth was 12.2 μm. When the grinding depth was 20 μm, the crack propagation depth was 18.9 μm. It can be known that increasing the grinding depth increased the crack propagation depth. The reason for this phenomenon may be that the increase in the grinding depth increased the action path of the grinding wheel on the workpiece in the grinding contact area, which led to an increase in the grinding contact arc length, grinding temperature, and grinding resistance [29]. Therefore, the crack propagation depth increased with the increase in the grinding depth.

Figure 5 shows the relationship between the feed rate *v_w_* and the crack propagation depth *h*. It can be seen from Figure 5 that when the wheel speed was 38 m/s, the grinding depth was 12 µm, the feed rate was 2500 mm/min, and the longitudinal crack propagation depth was 10.3 µm. When the feed rate was 5500 mm/min, the crack propagation depth was 18.0 μm. It is known that increasing the feed rate increases the crack propagation depth. The reason for this phenomenon may be that the increase in the feed rate increases the area of workpiece materials that participate in grinding per unit of time. The contact time between the grinding point on the workpiece surface and the grinding wheel is shortened. However, the thermal conductivity of the diamond grinding wheel is better than that of silicon nitride ceramics. In a short time, the depth of the heat transferred zone on the workpiece is shallow, and most of the heat is transferred into the grinding wheel, resulting in a lower grinding temperature [30]. It is not conducive to increasing the value of the fracture toughness of ceramic materials. Furthermore, increasing the feed rate leads to an increase in the cutting thickness of a single abrasive grain and grinding force [31]. Thus, the crack propagation depth increases with the increase in the feed rate due to the combined action of various factors.

### 3.2. Influence of Grinding Parameters on Residual Stress

**Figure 6:**
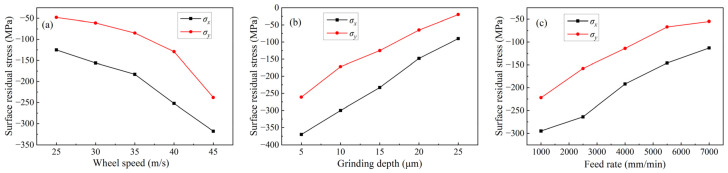
Surface residual stress under different grinding parameters: (**a**) Wheel speed; (**b**) Grinding depth; (**c**) Feed rate.

Figure 6 shows the residual stresses on the ground surface under different grinding parameters. According to Figure 6a, at a grinding depth of 12 µm and a feed rate of 3500 mm/min, when the wheel speed increased from 25 to 45 m/s, the surface residual stress in the direction parallel to the grinding direction increased from −125 to −318 MPa, and the surface residual stress in the direction perpendicular to the grinding direction increased from −48 to −238 MPa. A negative sign indicates that they are residual compressive stresses. According to Figure 6b, at a wheel speed of 38 m/s and a feed rate of 3500 mm/min, when the grinding depth increased from 5 to 25 µm, the surface residual stress in the direction parallel to the grinding direction decreased from −370 MPa to −90 MPa and the surface residual stress in the direction perpendicular to the grinding direction decreased from −261 MPa to −20 MPa. The residual stresses are also residual compressive stresses. According to Figure 6c, at a wheel speed of 38 m/s and a grinding depth of 12 µm, when the feed rate increases from 1000 to 7000 mm/min, the surface residual stress in the direction parallel to the grinding direction decreases from −295 to −113 MPa and the residual stress in the direction perpendicular to the grinding direction decreases from −222 to −55 MPa. The surface residual stresses are still residual compressive stresses. 

It can also be found from Figure 6 that no matter what kind of grinding parameters change, the value of residual stress in the direction parallel to the grinding direction is greater than that of the direction perpendicular to the grinding direction. The reason for this phenomenon may be that under the action of the grinding force, the abrasive grains in the grinding wheel carry out sliding, plowing, and cutting movements on the workpiece surface along the grinding direction during the grinding process, resulting in the extrusion effect along the grinding direction [32]. Therefore, it has a large value of residual stress in the direction parallel to the grinding direction compared to that in the direction perpendicular to the grinding direction.

### 3.3. Influence of Grinding Parameters on Grinding Temperature

**Figure 7:**
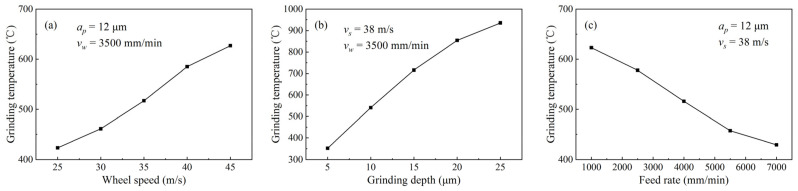
Grinding temperature under different grinding parameters: (**a**) Wheel speed; (**b**) Grinding depth; (**c**) Feed rate.

According to the experimental results under different grinding parameters, the variation curves of the grinding temperature with grinding parameters are shown in Figure 7.

According to Figure 7, at a grinding depth of 12 μm and a feed rate of 3500 mm/min, when the wheel speed increased from 25 to 45 m/s, the grinding temperature rose from 423 to 627 °C (Figure 7a), showing an increasing trend. At a wheel speed of 38 m/s and a feed rate of 3500 mm/min, when the grinding depth increased from 5 to 25 μm, the grinding temperature rose from 352 to 936 °C (Figure 7b), and also showed an increasing trend. At a grinding depth of 12 μm and a wheel speed of 38 m/s, when the feed rate increased from 1000 to 7000 mm/min, the grinding temperature decreased from 623 to 429 °C (Figure 7c), showing a decreasing trend. This reason is similar to the analysis in Section 3.1. The increase in the wheel speed increases the grinding power and heat flux density, and obstructs external convection heat transfer, leading to a tendency for the grinding temperature to rise. The increase in the grinding depth increases the grinding contact arc length, friction heat, and heat flux density so the grinding temperature has a tendency to increase. The increasing feed rate shortens the contact time between the grinding point on the workpiece surface and the grinding wheel, and most of the heat is transferred into the grinding wheel, resulting in a decreasing trend in grinding temperature.

In addition, the results can be found that the grinding depth has the greatest influence on the grinding temperature, followed by the wheel speed and the feed rate.

### 3.4. Influence of Grinding Temperature on Crack Propagation

**Figure 8:**
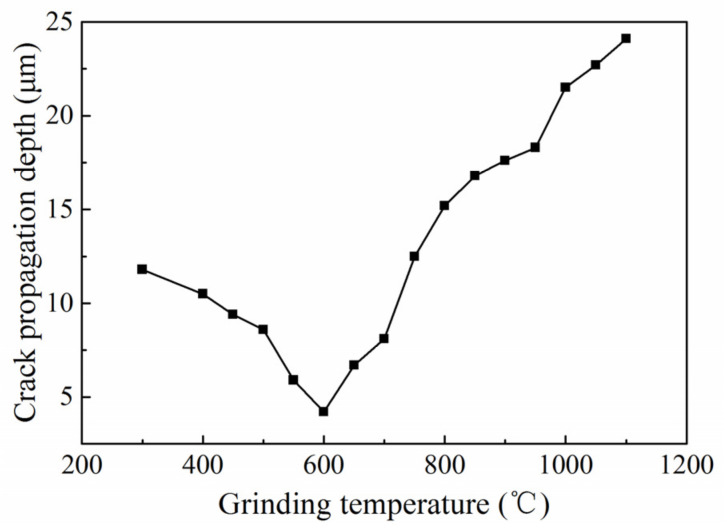
Crack propagation depth at different grinding temperature.

In order to analyze the influence of a single grinding temperature on crack growth, a set of experiments was added to adjust the grinding temperature by changing the coolant flow rate. The relationship between the grinding temperature and crack growth depth is shown in Figure 8. It can be seen from Figure 8 that the crack propagation depth decreased first and then increased when increasing the grinding temperature. With the grinding temperature rising from 300 to 1100 °C, the subsurface crack growth depth changed in scope from 4.2 to 24.1 μm. In a temperature range from 550 to 650 °C, the crack propagation depth had an approximation minimum value of 4.2 μm. The reason for this may be that an increase in the grinding temperature increases the heat flux density in the grinding area. The heat transferred to the workpiece also increases, which softens the glass phase on the surface/subsurface of the ceramic material, resulting in an increase in the fracture toughness of silicon nitride ceramics. According to Equations (1) and (2), increasing the grinding temperature in a certain range can increase the critical cutting depth of ductile-brittle transition and maximum undeformed chip thickness, resulting in an increase in the plastic grinding area. Then, materials on the surface of the workpiece are mostly removed by plastic deformation. The brittle fracture and spalling are reduced relatively. Therefore, crack propagation is inhibited. However, as the grinding temperature continues to rise, the temperatures on and inside the grinding surface have a large change, which generates thermal stress. The thermal stress, *σ_st_*, can be calculated by Equation (3) [23]. According to Equation (3), the greater the temperature change, the greater the hot-pressing stress is. When the value of hot-pressing stress exceeds the limit value of material fracture, hot cracks occur on the material surface, and the crack propagation depth increases with an increase in temperature. Therefore, the crack propagation depth decreases first and then increases with the increase in the grinding temperature, and there is an optimum grinding temperature for improving the surface quality in grinding.

(3) $$\sigma_{st}=\frac{E\alpha \Delta T}{1-\mu }$$

where *μ* is Poisson’s ratio of the material, and Δ*T* indicates the difference of temperature in the grinding surface.

## 4. Discussion

**Figure 9:**
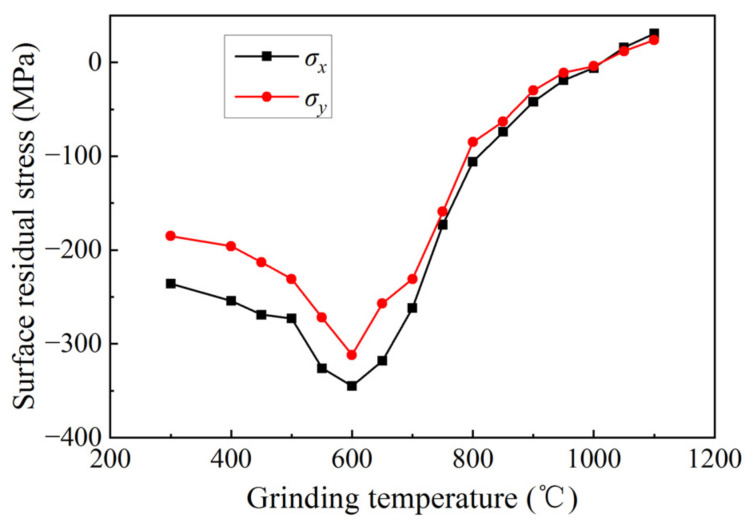
Surface residual stress at different grinding temperature.

Figure 9 displays the relationship between the surface residual stress and grinding temperature in the ground surface of silicon nitride ceramics. In Figure 9, when the grinding temperature is 600 °C, the value of residual compressive stress is the largest at −345 MPa in the direction parallel to the grinding direction and −312 MPa in the direction perpendicular to the grinding direction. When the grinding temperature is less than 600 °C, the residual compressive stress gradually increases with the increase in the grinding temperature. When the grinding temperature exceeds 600 °C, the residual compressive stress gradually decreases with the increase in the grinding temperature. When the grinding temperature reaches 1000–1050 °C, the residual compressive stress changes into tensile stress. The increase in the grinding temperature increases the fracture toughness of ceramic materials and reduces the brittleness removal of ceramic materials, resulting in an increase in the surface residual compressive stress of silicon nitride ceramics [33,34]. When the temperature exceeds a certain critical value, the effect of thermal stress makes the surface residual compressive stress decrease gradually. Under the action of grinding parameters and grinding temperature, the surface residual compressive stress even becomes residual tensile stress.

According to the above results, it was found that a certain range of grinding temperature could increase the surface residual compressive stress and inhibit the crack propagation on the surface/subsurface of silicon nitride ceramic materials. Therefore, the grinding temperature should be reasonably controlled in the grinding process.

**Figure 10:**
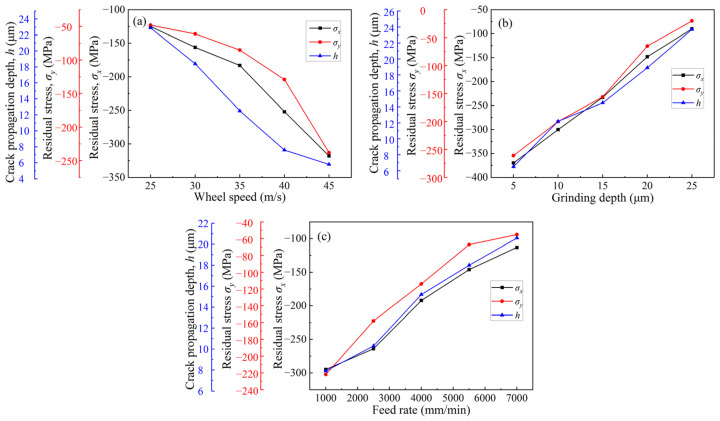
Surface residual stress and crack propagation: (**a**) Wheel speed; (**b**) Grinding depth; (**c**) Feed rate.

Figure 10 displays the relationship between residual stress and longitudinal crack propagation under different grinding parameters more intuitively. According to Figure 10a, the longitudinal crack propagation depth decreased from 22.9 to 5.8 µm with the increase in the surface residual compressive stress. As shown in Figure 10b,c, with the decrease in the residual compressive stress, the longitudinal crack propagation depth increased from 6.5 to 23.7 µm (Figure 10b) and 7.9 to 20.6 µm (Figure 10c), respectively. The reason for this phenomenon may be that when the wheel speed increases, it causes high temperature on the ground surface, which increases the value of the fracture toughness of ceramic materials, resulting in the materials tending to be removed mainly by elastic or plastic deformation [35,36,37]. The crack propagation and surface spalling are suppressed (Figure 10a). The stress on the ground surface cannot be released, and the surface residual stress increases. Therefore, the value of residual compressive stress increases and the crack propagation depth decreases. However, when the grinding depth and feed rate increase, more brittle spalling occurs on the ground’s surface [38]. The proportion of the plastic deformation-dominated removal method was greatly reduced. The value of surface residual compressive stress decreased. Meanwhile, the increase in the grinding depth and feed rate led to an increase in the surface/subsurface crack propagation depth (Figure 10b,c). The crack propagation causes the release of residual stress in ceramic materials and also leads to a decrease in the residual compressive stress value.

**Figure 11:**
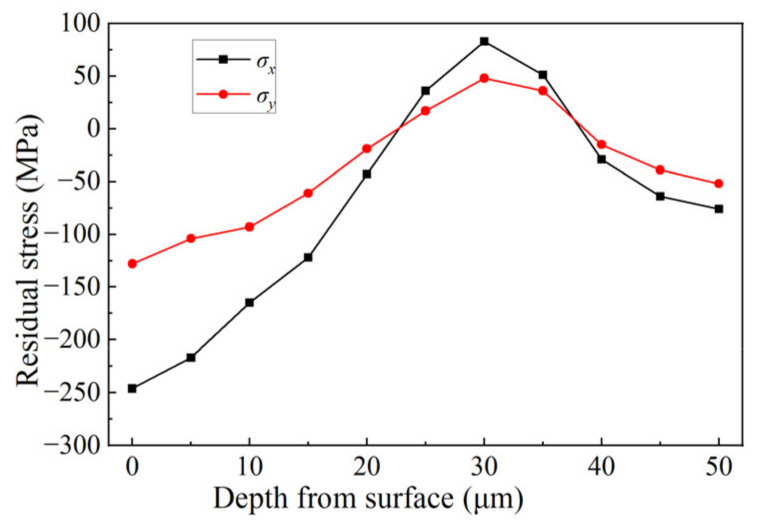
Distribution of residual stress in silicon nitride ceramic along depth direction.

Figure 11 shows the distribution of residual stress in silicon nitride ceramics (in a grinding parameter of vs. = 38 m/s, *a_p_* = 12 μm, *v_w_* = 3500 mm/min). According to Figure 11, the residual stresses along the grinding direction and perpendicular to the grinding direction are mainly compressive stress. At a distance from 20 to 25 μm from the surface to the inside of the workpiece, the compressive stress changes into tensile stress. The residual tensile stress is mainly distributed in the range from 25 to 35 μm below the grinding surface, and the other depth ranges are all compressive stress. In the direction perpendicular to the grinding direction, the peak value of residual compressive stress was −246 MPa on the grinding surface, and the peak value of residual tensile stress was 83 MPa. In the direction parallel to the grinding direction, the peak value of residual compressive stress was −128 MPa on the grinding surface, and the peak value of residual tensile stress was 48 MPa. Comparing the data of the two curves, it could be concluded that the variation range and peak value of the residual stress parallel to the grinding direction were greater than those perpendicular to the grinding direction. The reason may be that with the increase in the measuring depth, the effect of crack propagation became weak, and the residual compressive stress generated by the extrusion effect decreased [39]. The original residual compressive stress changed into tensile stress. In addition, the thermal stress produced by a high grinding temperature causes the state transition of grinding residual stress [40]. The large residual compressive stress changed to large residual tensile stress. With the increase in the measuring depth, the residual stress values in both directions gradually decreased, and the residual stress caused by grinding at a certain depth would be eliminated eventually.

**Figure 12:**
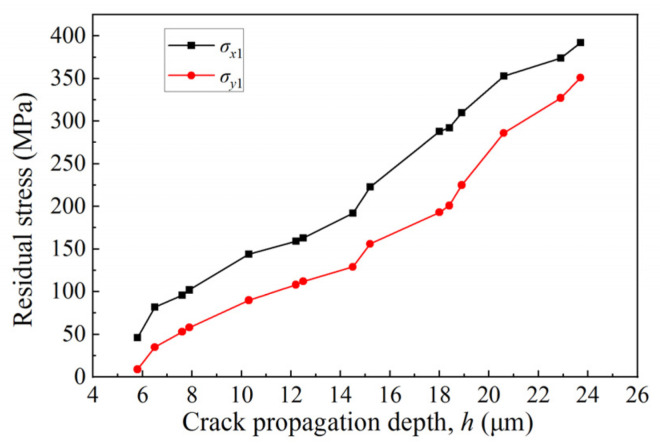
Relationship between residual stress at the location of crack propagation and crack propagation depth.

Since the residual stress value of the ground surface is the average value obtained from multiple points on the machined surface (9 mm × 3 mm), the final result is residual compressive stress. While the residual stress measured by sampling at the location of crack growth is residual tensile stress, and the rest is residual compressive stress. In order to explore the relationship between the surface residual stress at the location of crack growth and the crack propagation, the curves between the residual stress values (*σ_x_*_1_, *σ_y_*_1_) at the location of crack growth and the longitudinal crack propagation depth, *h*, are obtained under different grinding parameters, as shown in Figure 12. The specific parameters and results of the experiment are shown in Table 2. It can be seen from Figure 12 that the residual stress at the location of crack growth (parallel to the grinding direction and perpendicular to the grinding direction) is the residual tensile stress. The deeper the crack growth depth is, the greater the value of the residual tensile stress is. This result is consistent with the above analysis. The crack propagation and brittle spalling in the surface of the material will cause the release of the residual stress inside the ceramic material, which leads to a decrease in the residual compressive stress and gradually changes to the residual tensile stress. Then, with the increase in the crack propagation depth, the value of the residual tensile stress increases.

## 5. Conclusions

In this study, the effects of the grinding parameters on grinding temperature, surface residual stress, and crack propagation depth of silicon nitride ceramics were investigated. The relationships among the grinding temperature, surface residual stress and crack propagation depth were discussed. Based on the results, the following conclusions have been drawn:

(1) Increasing the wheel speed, reducing the grinding depth, and lowering the feed rate are beneficial to decreasing the depth of longitudinal crack propagation in the subsurface of silicon nitride ceramics. After grinding, the cracks in the subsurface of the silicon nitride ceramic workpiece are mainly longitudinal cracks, which gradually extend to the interior of the ceramics in multiple directions, resulting in subsurface damage.
(2) The residual compressive stress on the surface of silicon nitride ceramics increases with the increase in the wheel speed and the decrease in the grinding depth and feed rate. The value of residual compressive stress parallel to the grinding direction is greater than that perpendicular to the grinding direction.
(3) With the increase in the wheel speed and grinding depth and with the decrease in the feed rate, the grinding temperature has a tendency to increase. When the grinding temperature rises from 300 to 1100 °C, the surface residual compressive stress increases first and then decreases, and the crack propagation depth decreases first and then increases. At the temperature of about 600 °C, the surface residual compressive stress has a maximum value, and the crack propagation depth is the smallest. The proper grinding temperature can increase the surface residual compressive stress and inhibit the crack propagation of silicon nitride ceramics.
(4) The residual compressive stress on the ground surface of silicon nitride ceramics decreases with the increase in the crack propagation depth and surface brittle peeling degree. The residual stress at the location of crack propagation is residual tensile stress. It increases with the increase in the crack propagation depth. In addition, the distribution of residual stress alternates between compressive and tensile along the distance into surface, and this is eliminated at a certain depth.
(5) The surface quality of silicon nitride ceramics in grinding can be improved by adjusting the grinding parameters and controlling for an appropriate grinding temperature. The results can provide a useful reference to obtain high-quality machined surface in grinding of silicon nitride ceramics.

## Figures and Tables

**Table 1:** Properties of HIPSN ceramic materials.

Parameter	Value	Parameter	Value
Material characteristics	HIPSN	Compressive strength (MPa)	4500
Phase composition	*β*-Si_3_N_4_	Bending strength (MPa)	900
Compactness	98%	Fracture toughness (MPa·m^1/2^)	6
Density (kg/m^3^)	3240	Poisson’s ratio	0.26
Elastic modulus (GPa)	310	Hardness (GPa)	32
Thermal conductivity (W/(m·k))	32	Coefficient of thermal expansion (×10^−6^/K)	3.2

**Table 2:** Grinding experimental results.

Number	*v_s_* (m/s)	*a_p_* (μm)	*v_w_* (mm/min)	*σ_x_*_1_ (MPa)	*σ_y_*_1_ (MPa)	*h* (μm)
1–5	25, 30, 35, 40, 45	12	3500	374, 292, 163, 96, 46	327, 201, 112, 53, 9	22.9, 18.4, 12.5, 7.6, 5.8
6–10	38	5, 10, 15, 20, 25	3500	82, 159, 192, 310, 392	35, 108, 129, 225, 351	6.5, 12.2, 14.5, 18.9, 23.7
11–15	38	12	1000, 2500, 4000, 5500, 7000	102, 144, 223, 288, 353	58, 90, 156, 193, 286	7.9, 10.3, 15.2, 18.0, 20.6

## Data Availability

The data presented in this study are available in the article.
